# Novel Roles for the Sirtuin Deacylase SIRT5 in Normal Physiology and in Cancer

**DOI:** 10.1093/geroni/igaa057.2652

**Published:** 2020-12-16

**Authors:** David Lombard

**Affiliations:** University of Michigan, Ann Arbor, Michigan, United States

## Abstract

Sirtuins are NAD+-dependent deacylases that regulate diverse cellular processes such as metabolic homeostasis and genomic integrity. Mammals possess seven sirtuin family members, SIRT1-SIRT7, that display diverse subcellular localization patterns, catalytic activities, protein targets, and biological functions. Three sirtuins, SIRT3, SIRT4, and SIRT5, are primarily located in the mitochondrial matrix. SIRT5 is a very inefficient deacetylase, instead removing negatively charged post-translational modifications (succinyl, glutaryl, and malonyl groups) from lysines of its target proteins, in mitochondria and throughout the cell. SIRT5 plays only modest known roles in normal physiology, with its major functions occurring in the heart under stress conditions. In contrast, in specific cancer types, including melanoma, we have identified a major pro-survival role for SIRT5. We have traced this function of SIRT5 to novel roles for this protein in regulating chromatin biology. New insights into mechanisms of SIRT5 action in cancer, and in normal myocardium, will be discussed.

